# Altered mitochondrial function: a clue therapeutic strategies between metabolic dysfunction-associated steatotic liver disease and chronic kidney disease?

**DOI:** 10.3389/fnut.2025.1613640

**Published:** 2025-06-13

**Authors:** Jiang Bai, Lijuan Zhang, Letian He, Yun Zhou

**Affiliations:** ^1^Department of Nephrology, The First Hospital of Shanxi Medical University, Taiyuan, China; ^2^The First Clinical Medical School, Shanxi Medical University, Taiyuan, China; ^3^The Nephrology Department of Shanxi Provincial People’s Hospital, Shanxi Medical University, Taiyuan, China; ^4^The Fifth Clinical Medical School, Shanxi Medical University, Taiyuan, China

**Keywords:** metabolic dysfunction-associated steatotic liver disease, chronic kidney disease, mitochondrial dysfunction, oxidative stress, review

## Abstract

Metabolic dysfunction-associated steatotic liver disease (MASLD) and chronic kidney disease (CKD) have been demonstrated to be intricately linked in a multitude of research studies. The reclassification of MASLD has prompted a reevaluation of its epidemiological patterns and the associated risk of CKD. This is crucial as MASLD, focusing on cardiometabolic factors, might have a more pronounced association with CKD than NAFLD. Additionally, mitochondrial dysfunction has been implicated in the pathogenesis of both MASLD and CKD. Studies on metabolic dysfunction-associated steatohepatitis mouse models have revealed significant mitochondrial alterations, such as loss of cristae and impaired function in the kidneys, underscoring the critical importance of mitochondrial integrity in these pathologies. This review offers an extensive overview of the existing literature, covering the following key aspects: (a) presenting the latest epidemiological findings that elucidate the relationship between MASLD and CKD; (b) kidney pathological changes associated with MASLD; (c) mitochondrial alterations in MASLD and CKD, including oxidative stress, dynamics, and mitophagy; and (d) potential mitochondrial-targeted therapies.

## Introduction

Metabolic dysfunction-associated steatotic liver disease (MASLD) and chronic kidney disease (CKD) represent two major non-communicable diseases impacting global health. MASLD affects nearly 30% of the adult population (1), while CKD affects 8.2% (2). By the year 2031, it is projected that the incidence of MASLD will increase by 16.1% and that of CKD by 11.4% (3). This increase will significantly exacerbate the burden of these conditions, which are linked to poor prognosis, premature mortality, and reduced quality of life (4). A significant association exists between MASLD and CKD, characterized by notably lower glomerular filtration rates (GFR) among MASLD patients, indicating that MASLD may elevate the CKD risk (5, 6). Furthermore, in patients with MASLD, the proteinuria and the reduction in GFR are associated with the degree of hepatic fibrosis (7). Recent studies have introduced the term MLKD as an abbreviation to better encapsulate the relationship between MASLD and CKD (MLKD) (8).

**Figure 1:**
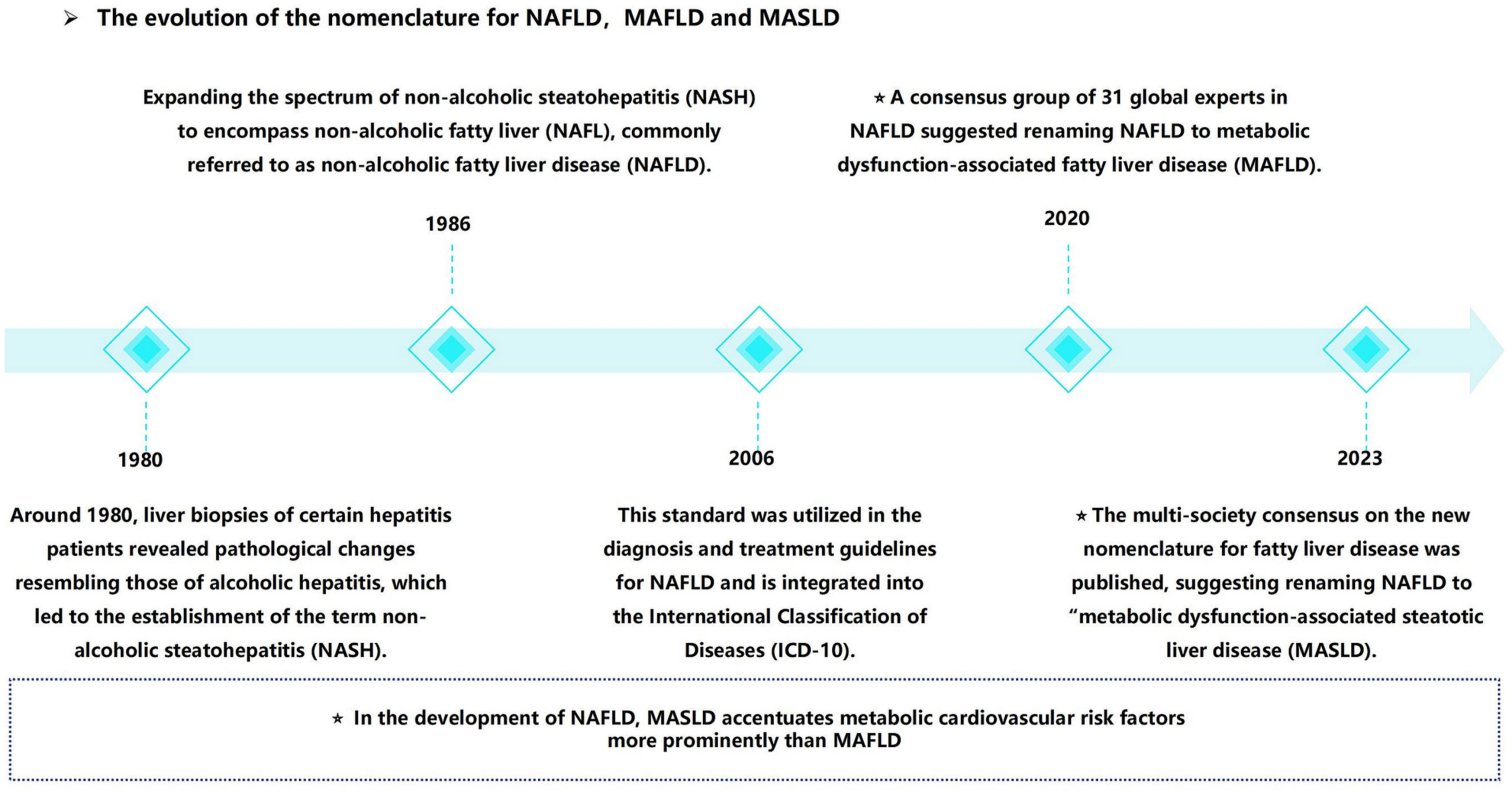
From non-alcoholic fatty liver disease (NAFLD) to metabolic dysfunction associated steatotic liver disease (MASLD), the naming evolution of MASLD.

In recent years, a notable shift has occurred in the field, marked by the reclassification from “non-alcoholic fatty liver disease” (NAFLD) to MASLD. Initially defined in the 1980s, non-alcoholic steatohepatitis (NASH) was later broadened to encompass NAFLD (9). In 2020, the term “metabolic dysfunction-associated fatty liver disease” (MAFLD) was proposed (10, 11), requiring hepatic steatosis in conjunction with a minimum of one metabolic risk factor: type 2 diabetes mellitus (T2DM), overweight/obesity, or evidence of metabolic dysfunction in individuals with lean or normal body weight (10). In 2023, “NAFLD” was redefined as “MASLD” (12). In contrast to the traditional definition, MASLD places increased emphasis on the metabolic and cardiovascular risk factors. CKD can emerge as a result of metabolic dysfunction. This implies that individuals with MAFLD or MASLD—manifested by hepatic steatosis in the presence of at least one metabolic factor—are at an increased risk of CKD (4) (Figure 1).

Establishing a causal relationship between MLKD proves challenging; however, numerous research indicate that mitochondrial dysfunction is a key factor in the development of both conditions. Mitochondria play a crucial role in cellular energy metabolism, influencing energy production, redox balance, and apoptosis (13–15). Recent studies on metabolic dysfunction-associated steatohepatitis (MASH) have demonstrated mitochondrial swelling and loss of cristae, underscoring the crucial involvement of mitochondrial dysfunction in MLKD (16). This review aims to address a critical gap in the current literature regarding the interrelationship between MLKD, a condition that has garnered increasing attention yet remains underexplored. By offering an extensive overview of MLKD’s epidemiology, the kidney pathological changes associated with MASLD, and the mechanisms linking mitochondrial dysfunction to MLKD development, this review seeks to elucidate these connections. Ultimately, we hope to identify effective mitochondrial-targeted therapies to address this growing health challenge.

## Epidemiological evidence of the association between MASLD and risk of CKD

Two studies published in 2008 (17, 18) first reported an increased risk of CKD among NAFLD patients, separate from typical factors associated with kidney dysfunction. Subsequent studies have further substantiated a notable correlation between NAFLD and the risk of CKD (4). An analysis synthesizing data included approximately 12 million middle-aged individuals, revealed that 28.1% of subjects in 13 longitudinal studies had NAFLD, which was associated with CKD (HR 1.43) (7). This analysis also revealed that the risk of CKD escalates as liver disease advances, particularly among those with more advanced liver conditions (7). The term “MAFLD” was introduced as a transitional term in 2020, while “MASLD” was formally adopted in 2023. Following the reclassification of MAFLD (10), several research investigated the influence of MAFLD on CKD risk, confirming an elevated risk of CKD among MAFLD patients (19–22).

As shown in Table 1, we focus on longitudinal studies from 2023 following the renaming to MASLD (12) that examine the association between MLKD. Heo et al.’s study including 57,785 Korean patients diagnosed with MASLD and with normal renal function at baseline, which revealed a CKD incidence of 11.31 per 10^4^ person-years (23). Compared to non-MASLD individuals, those with MASLD exhibited a higher CKD risk (HR 1.21). In this study, MASLD was found to more effectively identify CKD and proteinuria risk compared to NAFLD (23). Gao et al. (6) recruited 79,540 participants who were diagnosed with hepatic steatosis through ultrasound, of which 19,062 were identified as having MASLD. The CKD incidence rate among MASLD patients was 22.4 per 10^3^ person-years. After adjusting for confounders, MASLD exhibited a substantially elevated risk of CKD, with a HR of 1.15. The study also found that MASLD more effectively identifies CKD risk compared to conventional NAFLD, as demonstrated above (6). In a study by Mori et al. (24), which followed 3,187 Japanese patients with MASLD, the CKD incidence rate among MASLD patients was 28.3 per 10^3^ person-years (24). MASLD subjects had a significantly higher incidence risk of CKD (HR 1.20) compared to those with non-steatotic liver disease (non-SLD) (24). Therefore, numerous epidemiological studies suggest that the definition of MASLD effectively identifies high-risk subgroups of patients who are likely to develop CKD. Moreover, MASLD appears to be a superior indicator of CKD risk compared to conventional NAFLD. This underscores the critical necessity of recognizing and addressing MLKD as a significant public health concern, while also highlighting the imperative for early identification and intervention to effectively mitigate this risk. Given the observational nature of the existing studies, the causal link between MASLD and increased CKD incidence remains uncertain. Additionally, the aforementioned study was conducted on an Asian population, highlighting the need for future studies to investigate other regions or ethnicities.

**Table 1:** Longitudinal studies evaluating the relationship between MASLD and CKD following the adoption of the new MASLD nomenclature in 2023.

Author (References)	Country	Study characteristics	Follow-up period	Definition of SLD	Number of MASLD patients	Age (mean or median) (years)	Male, *n* (%)	Definition of CKD; Incidence of CKD	Covariate adjustments	HR (95% CI)
Heo et al. (23)	Korean	The cohort study of Korean individuals aged 18 years and older who received annual or biannual comprehensive health checkups at the Kangbuk Samsung Hospital Health Checkup Centers in South Korea. All study participants who underwent thorough physical examinations at least twice between January 1, 2011, and December 31, 2018, as well as those who had at least one additional follow-up visit before December 31, 2020, were included in the analysis	The mean follow-up time was 6.1 years	The presence of fatty liver was based on typical ultrasonographic features, such as hepatorenal echo contrast, liver brightness, or vascular blurring	57,785	39.8	50,521 (87.4)	CKD was defined as occurrence of eGFR < 60 mL/min/1.73 m^2^ over the follow-up period. Incidence of CKD was 11.31 (10.25–12.48) (per 10^4^ person years)	Age, sex, education level, smoking history, regular exercise (3 times/week), alcohol intake, prior history of coronary artery disease, use of any anti hypertensive medications, and eGFR at baseline	1.21 (1.04–1.42)
Gao et al. (6)	China	The Kailuan cohort was established in the Kailuan community of Tangshan City, Hebei Province, China. Between June 2006 and October 2007, they performed reexaminations at 2-year intervals up to the end of the last follow-up on December 31, 2019	The median follow-up time was 12.9 years	Hepatic steatosis was diagnosed by ultrasound	19,062	51.6	14,966 (78.5)	CKD was defined as eGFR <60 mL/min per 1.73m^2^ or positive proteinuria (≥1+). Incidence of CKD was 22.4 (21.8–23.0)(per 10^3^ person-years)	Age, sex, smoking habits, drinking consumption, exercise, education, income, baseline eGFR, uric acid, alanine aminotransferase, metabolic dysfunction, use of antihyperglycemic agents, use of antihypertensive agents, use of antilipidemic agents.	1.15 (1.11–1.19)
Mori et al. (24)	Japan	All individuals who received annual health examinations at Keijinkai Maruyama Clinic, Sapporo, Japan in 2006 were enrolled in this registry	The median follow-up time was 9 years	Steatotic liver disease was defined as the presence of hepatic steatosis determined by abdominal ultrasonography	3,187	49	2,655 (83.3)	CKD was defined as eGFR <60 mL/min/1.73 m^2^ or positive for urinary protein by the dipstick method. Incidence of CKD was 28.3 (per 10^3^ person-years)	Age, sex, eGFR, current smoking habit, diabetes mellitus, hypertension, and dyslipidemia	1.20 (1.08–1.33)
Jung et al. (5)	Korean	The retrospective, longitudinal, multicenter study included participants diagnosed with MASLD and who underwent TE between July 2006 and October 2018 at the Severance Hospital and Gangnam Severance Hospital of the Yonsei University Health System, two tertiary medical centers in Seoul, South Korea	The median follow-up time was 3.6 years	The presence of steatotic liver disease was defined as a CAP value ≥275 dB/m	3,240	51.7	1,899 (58.6)	CKD, de fined as an eGFR < 60 mL/min/1.73 m^2^ or proteinuria (≥1+ on dip stick test) on two consecutive measurements during follow-up. Incidence of CKD was 1.3 (1.1–1.4) (per 100 person-years)	Age, sex, and BMI, hypertension, dyslipidemia, use of anti-diabetic agents, baseline eGFR, fasting glucose, gamma glutamyl transferase, total cholesterol and triglycerides	NA

MASLD, metabolic dysfunction-associated steatotic liver disease; CKD, chronic kidney disease; SLD, steatotic liver disease; eGFR, estimated glomerular filtration rate; BMI, body mass index; CAP, controlled attenuation index; NA, not available.

### Kidney pathological changes linked to MASLD

#### Animal evidence

In various MASLD mouse models, including those subjected to high-fat diet (HFD) (25, 26) combined or not with weekly low-dose CCl4 (16), significant pathological damage to the kidneys has been documented. The HFD model is known for its capacity to induce metabolic disturbances, including obesity, insulin resistance, and dyslipidemia, which are central to the pathogenesis of MASLD (27). The combination of a high-fat diet with CCl4-induced liver injury provides a more comprehensive model that encompasses both metabolic and fibro-inflammatory aspects of the disease (28).

These injuries are characterized by glomerular enlargement, increased cross-sectional area of the glomeruli, a higher percentage of mesangial area, and renal fibrosis in both the tubules and interstitium (16, 25, 26). Electron microscopy reveals a marked loss of podocyte foot processes. However, Saito et al. (25) noted that the quantity of podocytes expressing WT-1 did not decrease, and they found no significant correlation between levels of proteinuria and the foot process loss. This suggests that factors beyond the loss of foot processes may contribute to the development of proteinuria (25). Mice subjected to a HFD exhibited a considerable accumulation of lipid droplets in the renal tubules, along with notable increases in neutral lipids in the interstitial cells of both the glomeruli and tubules (26).

#### Human correlates

Li et al. (16) reported a patient with a history of NASH who subsequently developed proteinuria. Similar to the pathological features of renal injury observed in the MASH mouse models, the pathology in this patient was characterized by focal segmental glomerulosclerosis, increased mesangial matrix, glomerular enlargement, and tubular interstitial fibrosis with inflammatory infiltration (16). A number of non-atrophic tubules contained intracellular lipid and protein absorption droplets. Some podocyte foot processes mildly diminished, while the thickness of the glomerular basement membrane seemed normal. Abundant lipid inclusions were observed in podocytes, mesangial cells, and tubular epithelial cells (TECs) (16).

### Mitochondrial structural alterations in MLKD

Mitochondria are organelles with double membranes that contain their own genetic material. Generally, their diameter ranges from approximately 0.5 to 1.0 μm, although size variations exist among different species. The outer mitochondrial membrane, which is smooth, functions as the boundary of the organelle. The inner mitochondrial membrane, characterized by its inward folds forming cristae, plays a crucial role in biochemical reactions (29).

The liver is particularly rich in mitochondria, with each liver cell containing between 1,000 and 2000 mitochondria (30, 31). Structural and functional impairments of mitochondria significantly contribute to the development of metabolic syndrome-related diseases (32–34). Patients with MASLD exhibit mitochondrial dysfunction characterized by mitochondrial swelling, cristae disorientation and fragmentation, mtDNA deletions, decreased activity of mitochondrial respiratory chain complexes, and impaired mitochondrial β-oxidation (35). As the disease progresses, mitochondrial mass increases, while the maximum respiratory capacity measured using high-resolution respirometry decreases by 31 to 40% (36). Alterations in mitochondrial structure, elevations in oxidative stress, and reductions in ATP production were more pronounced (37).

The kidneys are the second most energy-consuming organs, with more than 80% of renal oxygen consumption attributed to Na/K-ATPase activity. Glomerular cells predominantly depend on glucose for energy, resulting in comparatively low mitochondrial density (38–40). Renal tubular cells mainly rely on fatty acids (FAs) for energy and have a high mitochondrial content. The proximal convoluted tubules (PCT) lack the enzymes necessary for glycolysis and depend entirely on mitochondrial oxidation of key substrates including FAs, lactate, citrate, and glutamate to produce energy (41). Under pathological conditions, PCT cells exhibit depolarization of the mitochondrial membrane potential (MMP) (ΔΨm) (42, 43). Electron microscopy reveals mitochondrial damage, such as swelling and losing cristae. Notably, damaged mitochondria and lysosomes are observed surrounding myelin-like membranous inclusions within TECs (16). Mitochondrial abnormalities above have also been observed in individuals clinically diagnosed with MASH combined with CKD (16). This underlines the crucial involvement of the intricate and dynamic structure and functionality of mitochondria in the advancement of MLKD.

### Metabolic reprogramming of mitochondrial fatty acid oxidation

Under physiological conditions, tissues uptake FAs through various transport proteins, including scavenger receptor class B (CD36), fatty acid transport proteins (FATP), and fatty acid-binding protein (FABP). FATP are a class of multi-channel membrane proteins primarily responsible for transporting extracellular free fatty acids (FFAs) into cells while also participating in fatty acid metabolism (44). FABP, a family of intracellular proteins, recognizes long-chain fatty acids (LCFAs) as substrates, is important in the metabolism and transport of FAs (44). CD36 primarily facilitates the transport of LCFAs into cells. As a transmembrane glycoprotein, CD36 is widely expressed across various cell types, including adipocytes, hepatocytes, and TECs. It is involved not only in the uptake and transport of FAs but also plays a role in signal transduction (44–46).

Fatty acid oxidation is a crucial component of energy metabolism, crucial for maintaining energy balance and regulating lipid storage. Most FAs are oxidized within the mitochondria (44), with mitochondrial fatty acid oxidation (mtFAO) serving as essential pathway for reducing fat accumulation (47, 48). In the canonical pathway of mtFAO, FFAs are first activated to form acyl-CoA within the cytoplasm before being shifted into the mitochondrial matrix via carnitine palmitoyltransferase 1 (CPT1) and carnitine palmitoyltransferase 2 (CPT2) (45). Acyl-CoA is metabolized gradually by β-oxidation, the tricarboxylic acid (TCA) cycle, and oxidative phosphorylation (OXPHOS), producing a substantial amount of ATP.

**Figure 2:**
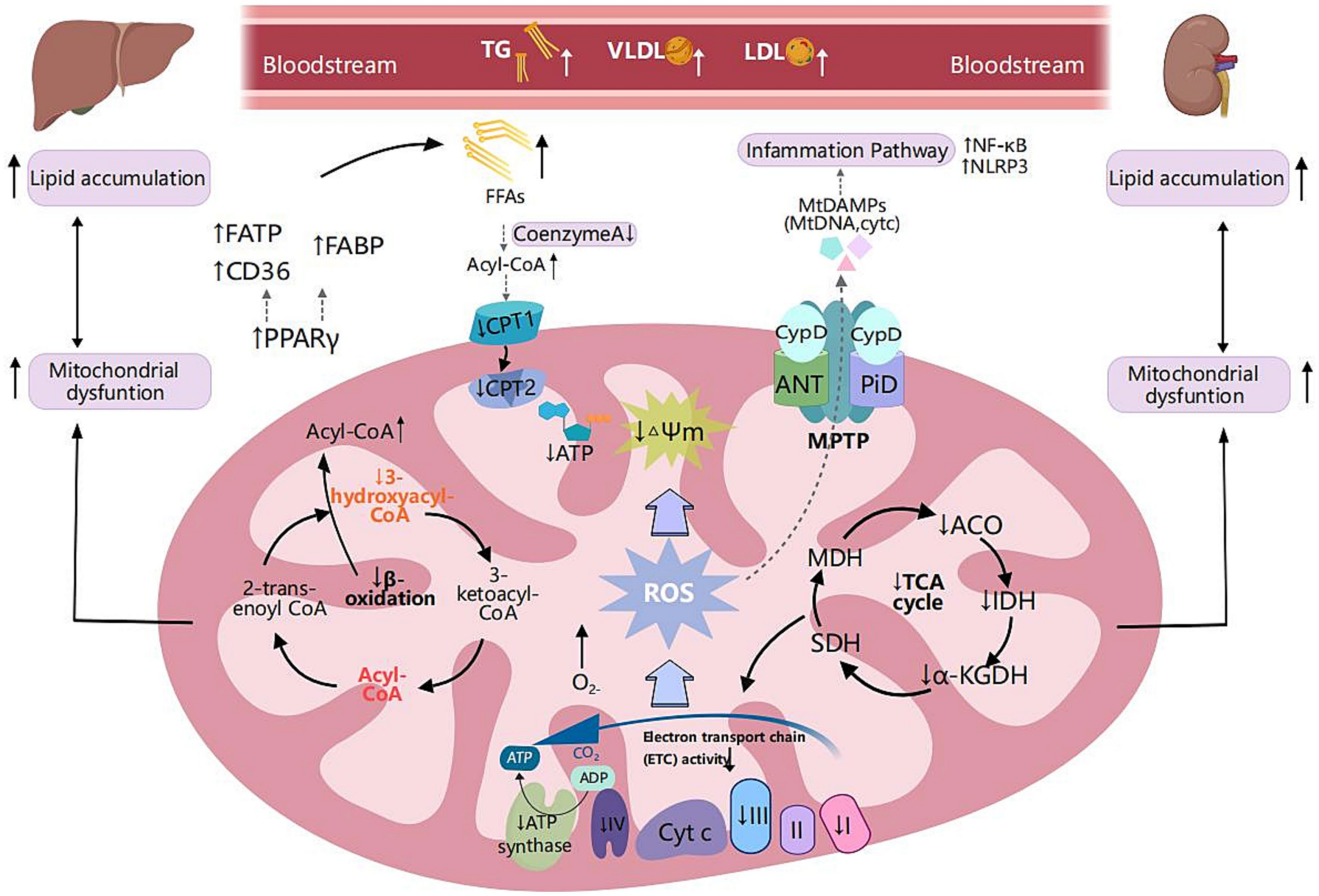
Metabolic reprogramming of mitochondrial fatty acid oxidation and shared pathogenic mechanisms in MASLD and CKD. Scavenger receptor class B (CD36), fatty acid transport proteins (FATP), and fatty acid-binding protein (FABP) are critical transport proteins involved in the uptake of fatty acids by tissues. Peroxisome proliferator-activated receptor-γ (PPAR-γ) is extensively expressed in adipose tissue, liver, and kidney. It regulates fatty acid transport proteins, and its upregulation is associated with lipid accumulation, inflammation, and fibrosis, thereby facilitating metabolic reprogramming. In MLKD, the upregulation of PPAR-γ correlates with elevated expression levels of CD36, FATP, and FABP, which correlates with free fatty acid (FFA) overload in renal and hepatic cells. Decreased levels of carnitine palmitoyltransferase 1 (CPT1) and carnitine palmitoyltransferase 2 (CPT2) lead to reduced fatty acid oxidation, insufficient production of NADH, decreased activity of the electron transport chain (ETC), and inadequate levels of ATP, ultimately resulting in mitochondrial dysfunction. Mitochondrial respiratory defects, specifically fatty acid oxidation (mtFAO) deficiency, can lead to chronic accumulation of FFAs and acyl-CoA, disrupting the function of the tricarboxylic acid cycle and mitochondrial respiration, leading to excessive production of reactive oxygen species (ROS). ROS can induce oxidative damage to mitochondrial DNA (mtDNA) and are associated with reduced mitochondrial membrane potential (MMP) and increased mitochondrial permeability exacerbating mitochondrial dysfunction, inflammation, and lipid peroxidation. Furthermore, ROS can disrupt the mitochondrial permeability transition pore (MPTP), resulting in the leakage of mtDNA into the cytoplasm. ROS trigger downstream signaling pathways regulating inflammatory responses, including the nuclear factor kappa B (NF-κB) pathway and the nucleotide-binding oligomerization domain-like receptor 3 (NLRP3) inflammasome pathway. This activation results in the production of substantial amounts of ROS, creating a vicious cycle of inflammation and oxidative damage. Created with MedPeer (medpeer.cn).

Peroxisome proliferator-activated receptor-γ (PPAR-γ) is extensively expressed in adipose tissue, liver, and kidneys. PPAR-γ regulates transport proteins, and its upregulation correlates with lipid accumulation, inflammation, and fibrosis, thereby promoting metabolic reprogramming and the development of MLKD (Figure 2). In the liver, PPAR-γ influences the uptake and metabolism of FAs through promoting the expression of transport proteins such as CD36 (49). Selective deletion of PPAR-γ significantly reduces hepatic steatosis caused by a HFD in hepatocytes (50). Hepatic steatosis induced by a HFD in mice is closely associated with the expression of CD36, FATP2, and FATP5 (51, 52). Liver cell-specific deletion of CD36 can mitigate HFD-induced fatty degeneration in mice (53). Within the nephron, proximal renal tubules and glomeruli are particularly vulnerable to lipid accumulation, leading to renal damage. This susceptibility is linked to the upregulation of PPAR-γ and lipid uptake transporters such as CD36 and FABP (41). Elevated activity of FATP1, FATP4, and FATP2 results in an overload of FFAs in renal cells (44).

Mitochondrial fatty acid oxidation respiratory deficiencies might significantly contribute to the progression of MLKD (Figure 2). Among the enzymes involved, β-hydroxyacyl-CoA dehydrogenase is particularly crucial in the β-oxidation pathway (54). Mary et al.’s study revealed a significant 40–50% reduction in β-hydroxyacyl-CoA dehydrogenase activity among MASLD patients compared to the control group, suggesting impaired β-oxidation (55). Furthermore, multiple studies have consistently shown decreased levels of CPT1 and CPT2 in animal models induced by a HFD (56–58). In obesity-related kidney disease models, the downregulation of long-chain acyl-CoA synthetase-1 (ACSL1), a vital enzyme in fatty acid oxidation, is closely linked to heightened lipid accumulation in the kidneys (54). Notably, in CKD, the diminished expression of CPT1 and CPT2 also results in decreased fatty acid oxidation, inadequate NADH generation, impaired electron transport chain (ETC) activity, depleted ATP levels, culminating in mitochondrial dysfunction (59).

The prolonged presence of elevated FFAs and persistent buildup of acyl-CoA disrupt the TCA cycle and mitochondrial respiration, leading to excessive production of reactive oxygen species (ROS) (60). Moreover, compromised mitochondrial β-oxidation can prompt the peroxisomal and cytosolic oxidation of FFAs, giving rise to peroxidative byproducts and excess ROS levels (61). ROS can induce oxidative damage to mitochondrial DNA (mtDNA), resulting in a reduction in MMP and an increase in mitochondrial permeability (30), exacerbating mitochondrial dysfunction and lipid peroxidation, thereby establishing a vicious cycle that favors metabolic reprogramming.

### Oxidative stress

ROS are generated from various pathways within the mitochondria, involving a series of interconnected mechanisms. Initially, electrons leak from complexes of the ETC and interact with oxygen to produce superoxide radicals. Subsequently, these superoxide produce other ROS, including superoxide anions and hydrogen peroxide (H₂O₂), through both enzymatic and non-enzymatic reactions. Dysfunction in the ETC or mitochondrial impairment can exacerbate this process, leading to an increased production of ROS (15, 62). The mitochondria possess several antioxidant defense mechanisms to mitigate ROS accumulation. Key antioxidant enzymes such as superoxide dismutase (SOD) and catalase, play crucial roles in ROS clearance (15, 63). Under physiological conditions, these enzymes convert superoxide radicals into H₂O₂ and water (64). A decline in antioxidant enzyme levels is a significant factor in the development of excessive oxidative stress.

As previously mentioned, lipid metabolism is a key factor in the oxidative stress associated with MLKD. Numerous studies have indicated that MASH patients exhibited elevated ROS (65–67). Additionally, some studies have confirmed an increase in mitochondrial ROS (mtROS) in CKD (68, 69), with FAs contributing to elevated ROS levels. A HFD increases CD36 expression in the kidneys of mice, while palmitic acid treatment enhances CD36 levels in podocytes *in vitro* (44). In contrast, treatment with the CD36 inhibitor has been shown to effectively reduce lipid accumulation and ROS production in podocytes (44, 70).

Elevated levels of ROS exacerbate oxidative stress associated with mitochondrial dysfunction, leading to cellular damage and lipid peroxidation, which further aggravate the pathological state of MASLD (15) and the progression of CKD (14, 71) (Figure 2). Additionally, ROS can disrupt the mitochondrial permeability transition pore (MPTP), resulting in the leakage of mtDNA into the cytoplasm (72). ROS triggers downstream signaling pathways that regulate inflammatory responses, leading to increased production of pro-inflammatory cytokines such as tumor necrosis factor-alpha (TNF-α) (62, 73). TNF-α exacerbates oxidative damage and inflammatory responses while triggering the activation of mitogen-activated protein kinases (MAPKs). This results in the generation of substantial amounts of ROS, especially superoxide anions. The consequent oxidative stress not only exacerbates damage to cellular components but also stimulates the generation of additional TNF-α, perpetuating a cycle of inflammation and oxidative damage (62, 73).

### Mitochondrial biogenesis

Mitochondrial biogenesis is a sophisticated process that enables cells to produce new mitochondria in order to satisfy their energy requirements and preserve cellular homeostasis. Mitochondrial transcription factor A (TFAM) is a crucial regulatory factor for mtDNA processes. By binding to mtDNA, TFAM promotes the production of mtDNA and proteins, transcribing and packaging them into nucleoids (74). The expression of TFAM is regulated by peroxisome proliferator-activated receptor-γ coactivator 1α (PGC-1α), which connects the nuclear control of mitochondrial biogenesis with the maintenance and expression of mtDNA (14). AMP-activated protein kinase (AMPK) facilitates the activation of PGC-1α (75, 76).

**Figure 3:**
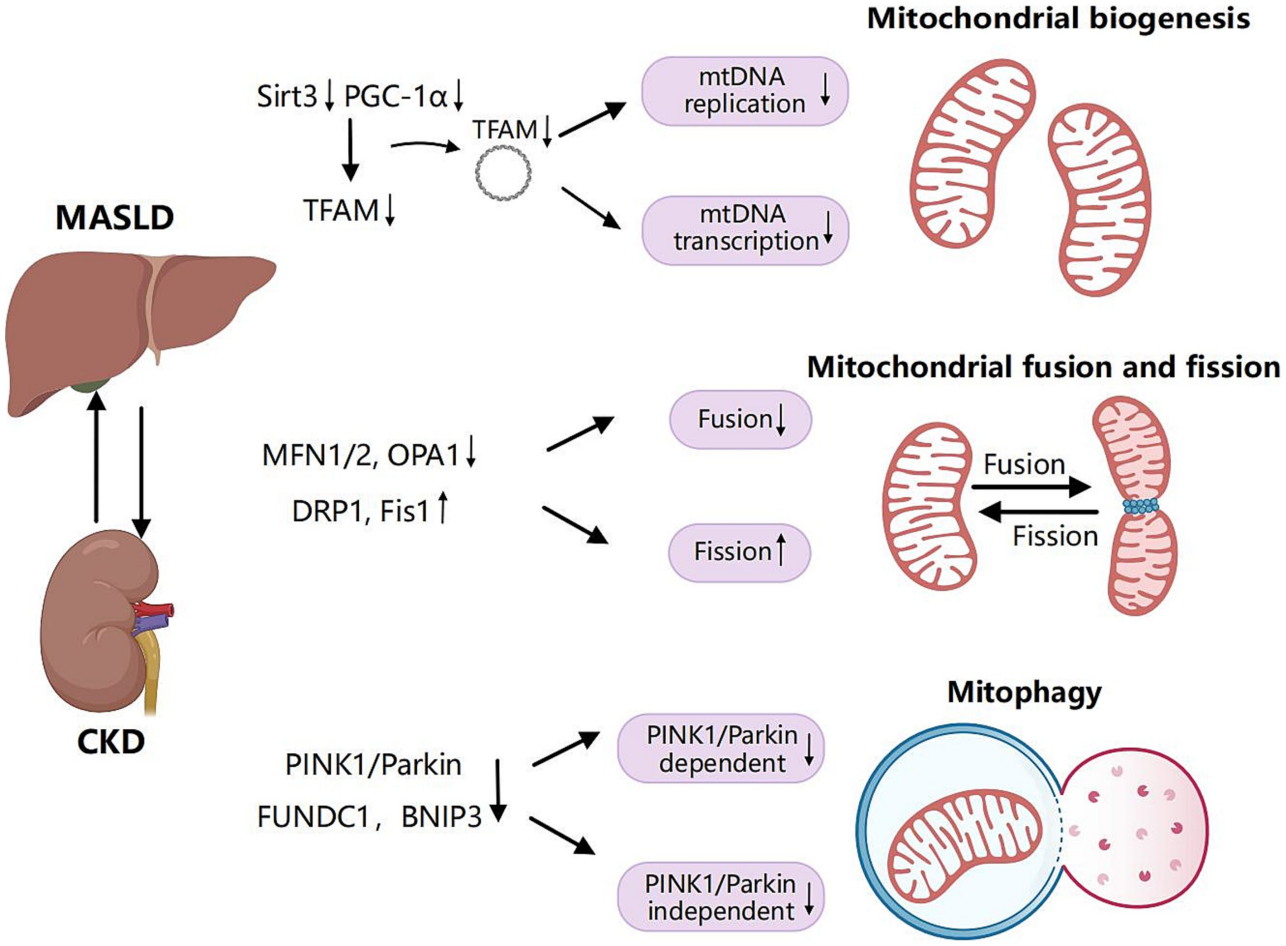
Shared pathogenic mechanisms of mitochondrial biogenesis, dynamics and mitophagy in MASLD and CKD. Mitochondrial biogenesis is a complex process through which cells generate new mitochondria to meet their energy demands and maintain cellular homeostasis. Mitochondrial transcription factor A (TFAM) serves as a pivotal regulatory factor in the transcription and replication of mitochondrial DNA (mtDNA). The expression of TFAM is regulated by peroxisome proliferator-activated receptor-γ coactivator 1α (PGC-1α). Inhibition of PGC-1α and sirtuin 3 (SIRT3) impedes mitochondrial biogenesis, resulting in a reduction of mitochondrial quantity and impaired functionality during the progression of MLKD. Mitochondrial fusion refers to the process by which individual mitochondria within a cell merge through the fusion of their membranes and contents. In contrast, mitochondrial fission involves a single mitochondrion dividing into two or more smaller mitochondria. Key proteins involved in mitochondrial fusion include mitofusin 1 (MFN1), mitofusin 2 (MFN2), and optic atrophy 1 (OPA1). Mitochondrial fission is primarily regulated by dynamin-related protein 1 (DRP1) and mitochondrial fission protein 1 (Fis1). Decreased expression of MFN1, MFN2, and OPA1 in the liver and kidney, combined with the upregulation of DRP1 and Fis1 in MLKD, results in the inhibition of mitochondrial fusion and an increase in mitochondrial fission. Mitophagy is a specialized form of autophagy that specifically targets damaged or dysfunctional mitochondria to remove them and prevent the accumulation of harmful components. This process plays a crucial role in maintaining mitochondrial quality control and cellular homeostasis. Mitophagy encompasses ubiquitin-dependent pathways, including those involving phosphatase and tensin homolog (PTEN)-induced putative kinase 1 (PINK1) and Parkin. Additionally, there are ubiquitin-independent pathways that involve receptors such as Bcl-2/adenovirus E1B 19-kDa protein-interacting protein 3 (BNIP3) and FUN14 domain-containing protein 1 (FUNDC1). A reduction in mitophagy-related proteins from both ubiquitin-dependent and independent pathways, including PINK1, Parkin, BNIP3, and FUNDC1, results in decreased mitophagy efficiency, exacerbating liver and kidney injury in MLKD. Created with MedPeer (medpeer.cn).

In MASLD, the expression of key regulatory factors involved in mitochondrial biogenesis (77), such as Sirt3 and PGC-1α, is diminished. In models of CKD in mice, levels of PGC-1α (78, 79) and SIRT3 (80) are consistently reduced. The downregulation of SIRT3 results in the hyperacetylation of mitochondrial proteins, thereby promoting oxidative stress and fat accumulation in the liver (77). Additionally, low levels of PGC-1α are linked to a decline in antioxidant mechanisms (81). Inhibition of PGC-1α impedes mitochondrial biogenesis, leading to a decrease in mitochondrial quantity and impaired functionality, which may ultimately result in cellular energy metabolism disorders and apoptosis. Overall, mitochondrial biogenesis is suppressed during the progression of MLKD (Figure 3).

### Mitochondrial dynamics: fusion and fission

Mitochondrial fusion is the process where individual mitochondria within a cell merge through the fusion of their membranes and contents. In contrast, mitochondrial fission refers to the division of one mitochondrion into multiple smaller units (14). Mitochondrial fusion is regulated by key proteins such as mitofusin 1 (MFN1) and mitofusin 2 (MFN2) on the outer mitochondrial membrane, which promote the connection and fusion of adjacent mitochondria, and optic atrophy 1 (OPA1) on the inner mitochondrial membrane, which controls inner membrane fusion (82). Mitochondrial fission is primarily regulated by dynamin-related protein 1 (DRP1) and mitochondrial fission protein 1 (Fis1). DRP1, a GTPase, forms spiral-like structures at the regions of mitochondrial constriction (15). Through mitochondrial fusion and fission, cells dynamically regulate the morphology and distribution of mitochondria to adapt to changing metabolic demands.

In patients with MASH, liver biopsies have shown a reduction in MFN2 levels (83). A HFD leads to a reduction in the levels of MFN1, MFN2, and OPA1. Concurrently, the expression of DRP1 is upregulated (84–86). FFA can increase the expression of Drp1 and Fis1 while reducing the expression of OPA1 and Mfn2 (87). The role of lipids in mitochondrial dysfunction associated with CKD has also received research support. Studies indicate that a HFD reduces the expression of MFN2 and OPA1 in the kidneys while increasing DRP1 expression (84, 88). Ko et al. (89) reported that in hypertensive kidney disease (HKD) rats, the expression of DRP1 was upregulated, whereas the expression of Mfn2 was downregulated. Acyl-CoA:lysocardiolipin acyltransferase-1 (ALCAT1) that regulates cardiolipin biosynthesis in mitochondrial. In a diabetic kidney disease (DKD) mouse model, the expression of MFN2 and OPA1 are diminished, whereas those of Drp1 and Fis1 are increased. Overexpression of ALCAT1 can enhance MFN2 and OPA1 expression while reducing Drp1 levels, which further mitigates the decline in estimated glomerular filtration rate (eGFR), thereby improving mitochondrial dynamics (90).

Disruption in the balance of mitochondrial fusion and fission impairs mitochondrial dynamics, leading to dysregulation of cellular homeostasis and mitochondrial function. As previously noted, key regulatory factors of mitochondrial fission and fusion include MFN, OPA1, DRP1, and Fis1. Alterations in the levels or function of these essential proteins disrupt the equilibrium between mitochondrial fission and fusion, leading to impaired mitochondrial structure and function, which in turn triggers inflammatory responses through various pathways (91, 92). Furthermore, mitochondrial dynamics are closely associated with cell death, particularly apoptosis, as some proteins linked to mitochondrial dynamics (e.g., Drp1 and Mfn2) directly regulate apoptotic processes (93, 94). The imbalance in mitochondrial dynamics, characterized by enhanced fission and suppressed fusion, leads to increased mitochondrial fragmentation, elevated permeability of the outer mitochondrial membrane, cytochrome leakage, and activation of caspases, ultimately inducing apoptosis (95) (Figure 3).

### Mitophagy

Mitophagy, a specialized autophagic process, selectively targets and removes damaged or dysfunctional mitochondria to prevent the accumulation of harmful components (15). It encompasses ubiquitin-dependent pathways, including phosphatase and tensin homolog (PTEN)-induced putative kinase 1 (PINK1) and Parkin. Additionally, there are ubiquitin-independent pathways involving receptors such as Bcl-2/adenovirus E1B 19-kDa protein-interacting protein 3 (BNIP3), FUN14 domain-containing protein 1 (FUNDC1), and cardiolipin (96, 97). Dysregulation of mitophagy leads to the accumulation of damaged mitochondria, further exacerbating mitochondrial dysfunction and contributing to the pathogenesis of MLKD. Disruptions in mitochondrial dynamics, such as imbalances in mitochondrial fusion and fission, can also affect mitophagy (98). These pathways ultimately form autophagolysosomes, facilitating the removal of damaged mitochondria (99).

Mitophagy is essential for regulating mitochondrial biogenesis and facilitating lipid β-oxidation within mitochondria (100). Conversely, impaired autophagy of damaged mitochondria fails to sustain an adequate number of healthy mitochondria, thereby contributing to hepatic steatosis and the progression of MASLD (101, 102). In experimental models of MASLD induced by a high-fat/high-calorie diet, the expression of PINK1 and Parkin is decreased, resulting in reduced mitophagy efficiency, exacerbating liver injury and ultimately progressing to MASH (37, 103). Insufficient FUNDC1-dependent mitophagy can drive the transition to HFD-induced MASLD (104). Data from Li R et al. indicated that liver injury induced by a HFD was linked to reduced Sirt3 expression, followed by inactivation of the ERK-CREB signaling and suppression of mitophagy driven by BNIP3, leading to mitochondrial-dependent cell death in hepatocytes (105).

Similarly, studies demonstrated that a HFD caused downregulation of PINK1 and Parkin expression in murine kidneys (106), resulting in impaired mitophagy, promoting mitochondrial injury in TECs, reducing MMP, and inducing apoptosis (107). In STZ-induced diabetic animal models, mitophagy-associated proteins from both ubiquitin-independent and non-independent pathways, such as FUNDC1, BNIP3 and PINK1 are reduced (98, 108). In a rat model of HKD established via 5/6 nephrectomy combined with DOCA-salt treatment (25 mg/kg, subcutaneous injection, twice weekly), the expression of PINK1/Parkin was upregulated (89).

The decreased stimulation of these pathways impairs the selective degradation of impaired mitochondria. Furthermore, the accumulation of dysfunctional mitochondria can result in elevated ROS production and intensified cellular stress, promoting persistent inflammation. Mitochondrial injury can trigger the release of mtDNA into the cytoplasm, which the immune mechanism recognizes as injury-associated molecular patterns (DAMPs). This recognition further promotes inflammatory pathways and amplifies the inflammatory response (109) (Figure 3).

### Mechanisms linking liver damage in MASLD to CKD development

While mitochondrial dysfunction has been implicated, other critical factors such as the activation of the renin-angiotensin-aldosterone system (RAAS), alterations in the gut microbiota, inflammation, and genetic predisposition likely also contribute. In MASLD, the liver, being the primary site for angiotensinogen synthesis, experiences increased production and release of angiotensinogen into the bloodstream due to its inflammatory and oxidative stress states. Angiotensinogen is subsequently converted to angiotensin I by renin and further to the potent vasoconstrictor angiotensin II by angiotensin-converting enzyme (ACE). Angiotensin II exacerbates liver injury by increasing intrahepatic resistance and elevates blood pressure through systemic vasoconstriction, emerging as a key risk factor for CKD development. Long-term excessive activation of the renin-angiotensin-aldosterone system (RAAS) induces renal ischemia, impairs renal filtration and electrolyte balance regulation, increases glomerular filtration membrane permeability, leading to proteinuria, and ultimately results in glomerulosclerosis and fibrosis, further weakening kidney function (110). Moreover, the vicious cycle initiated by RAAS activation in MASLD, driven by worsening liver injury and intensified RAAS activation, exacerbates renal ischemia and injury. The kidneys’ compromised ability to regulate fluid balance and blood pressure further stimulates RAAS activation, accelerating the progression of both diseases (111).

Intestinal microbiota imbalance is a hallmark pathological feature of both MASLD and CKD, significantly contributing to the pathogenesis of these conditions. In individuals with MASLD and CKD, the gut microbiota is characterized by reduced bacterial richness and diversity. Specifically, beneficial bacteria such as Lactobacillus and Bifidobacterium are diminished, while potentially pathogenic taxa like Enterobacteriaceae and Enterococcus are markedly enriched (8). This dysbiosis is closely associated with the disruption of intestinal epithelial tight junctions, leading to increased intestinal permeability. Consequently, lipopolysaccharide (LPS) from the gut can translocate into the systemic circulation. LPS, a potent immune activator, triggers signaling pathways involving NF-κB and TLR2 and TLR4. This activation exacerbates inflammation in the liver and kidneys, thereby accelerating the progression of hepatic fibrosis and renal fibrosis. Moreover, intestinal microbiota imbalance is also closely associated with the production and metabolic changes of various intestinal metabolites, which play a significant role in the development of MASLD and CKD (112).

Genetic factors significantly influence the development and progression of MASLD and CKD, with various genetic polymorphisms and mutations potentially linking these two conditions. For instance, the I148M SNP in the PNPLA3 gene is strongly associated with increased hepatic fat accumulation and liver injury, predisposing carriers to severe liver disease. Research revealed that PNPLA3 mRNA and protein are expressed not only in the liver but also in the kidneys, particularly in renal tubular cells and podocytes (113). Podocyte activation during injury can drive renal fibrosis and glomerulosclerosis. The PNPLA3 GG variant may exacerbate injury and promote ectopic lipid accumulation under conditions of lipid excess, potentially leading to lipid nephrotoxicity by influencing podocyte activation (114).

### Therapeutic strategies targeting mitochondria in MLKD

Given the significant overlap in pathogenesis between MLKD, treatment for one condition is likely to be effective for the other. As research on the association mechanism between MLKD progresses, some scholars have proposed the cardiac-kidney-liver (CKL) syndrome (115) or a framework referred to as the cardiovascular-renal-hepatic-metabolic (CRHM) syndrome (116). This management approach for heart, liver, and kidney functions by cardiology, endocrinology, nephrology, and hepatology is transitioning toward a more integrated model. This review focuses on the enhancement of oxidative stress mitigation, metabolic reprogramming, and mitochondrial homeostasis facilitated by antioxidants, sodium-glucose cotransporter-2 (SGLT2) inhibitors, glucagon-like peptide-1 (GLP-1) receptor agonists, PPAR-γ agonists, and mesenchymal stem cells (MSCs), along with their therapeutic roles in the management of MLKD.

### Antioxidants

In patients with MLKD, elevated levels of ROS and decreased plasma antioxidant activity are observed in both the liver and kidneys. ROS-induced oxidative stress is recognized as one of the essential mechanisms underlying the onset and progression of MLKD, and multiple studies have emphasized the beneficial effects of antioxidants in treatment. Mitochondrial-targeted drugs, such as mitoTEMPO, elamipretide (SS-31) and mito-quinone (Mito-Q), have demonstrated potential therapeutic benefits in various diseases associated with mitochondrial dysfunction, including MLKD (117, 118).

### Mito-Q

Mito-Q is synthesized by covalently linking ubiquinone, an intrinsic electron carrier from the ETC, with the lipophilic molecule triphenylphosphine (TPP). Within cells, Mito-Q achieves high concentrations, approximately 100 times those found in the cytosol (117). As an antioxidant targeting mitochondria, Mito-Q acts a critical role in mitigating oxidative stress in mitochondria, stimulating mitochondrial biogenesis, and facilitating mitophagy. These processes are mediated through AMPK and its downstream signaling pathways, including mTOR, NF-κB, Nrf2, and SIRT1, ultimately contributing to the alleviation of symptoms associated with metabolic syndrome, such as obesity and insulin resistance (119).

Mito-Q has been shown to exert protective effects in rats fed a HFD, leading to reductions in body weight, hepatic steatosis, blood lipid levels, and insulin levels (120, 121). This effect correlates with elevated levels of mitochondrial cardiolipin content, thereby enhancing the activity of complexes II, III, and V (121). Furthermore, a phase II clinical trial targeting chronic hepatitis C revealed that Mito-Q can reduce hepatic transaminase levels, indicating its protective role against necrotizing liver inflammation (122). In kidney, Mito-Q reverses the changes in podocyte mitochondrial morphology and function induced by angiotensin II stimulation, including decreased MMP, excessive production of ROS, and ATP deficiency (123). In a recent pilot randomized controlled trial, Kirkman DL et al. revealed that Mito-Q enhanced macrovascular endothelial function, arterial hemodynamics, and microvascular function, partly by decreasing NADPH oxidase-mediated vascular dysfunction in CKD patients (118).

### MitoTEMPO

MitoTEMPO is a mitochondrial-targeted antioxidant comprising the piperidine nitroxide (TEMPO) and TPP. TEMPO functions as a SOD mimic, capable of scavenging superoxide anions and alkyl radicals (117). In HFD-induced mice, MitoTEMPO improves liver lipid accumulation, alleviates inflammatory responses, and downregulates fibrosis-related gene expression (124). Moreover, MitoTEMPO inhibits ROS production, increases intrahepatic CD4^+^ T lymphocyte counts, and delays the development of hepatocellular carcinoma (HCC) induced by MASLD (125). Additionally, in human podocytes (HPC), MitoTEMPO stabilizes the MMP, while also mitigating the activation of the NLRP3 inflammasome through the PINK1/Parkin-mediated autophagy pathway, thereby improving podocyte injury (126). Finally, mitoTEMPO significantly decreases markers of mitochondrial dysfunction, and levels of pro-fibrotic factors in mice with CKD, thereby enhancing renal function and mitigating renal fibrosis (127).

### SS-31

Peptides targeting mitochondria, like Szeto-Schiller peptide 31 (SS-31), also known as elamipretide, are antioxidant peptides that selectively accumulate in the inner mitochondrial membrane (128, 129). Mechanistic studies demonstrate that SS-31 functions as a mitochondrial-targeted scavenger of mtROS, effectively mitigating oxidative stress while protecting cardiolipin from oxidative damage (90, 130, 131). In a mouse model of T2DM, SS-31 effectively prevents hepatic mitochondrial dysfunction by enhancing H₂O₂ metabolism, reducing lipid peroxidation and boosting ATP synthesis (132). Moreover, research by Hao et al. affirmed that SS-31 exhibited significant protective effects in mouse models of DKD by limiting cardiolipin oxidation and protecting podocytes (90). Szeto-schiller peptide 20 (SS-20) also demonstrates significant renal protective effects by reducing mtROS production and inflammatory responses (133).

### SGLT2 inhibitors

Recent research on the interaction between MLKD has brought SGLT2 inhibitors into the spotlight as a promising therapeutic option. Honda et al. (134) demonstrated that ipragliflozin upregulates genes associated with fatty acid β-oxidation and lipid export in the liver, thereby accelerating hepatic lipid metabolism and decreasing liver lipid content in MASH mice, which alleviated liver steatosis. Dapagliflozin can reverse the decline in mtDNA copy number in the livers of diabetic mice while increasing the levels of Mfn2, Drp1and PGC1α. This process normalizes mitochondrial respiration control in hepatocytes, reduces lipid peroxidation, and prevents the activation of the MPTP (135). In human proximal tubular cells (HK-2), Zaibi N et al. measured ROS production in the cytoplasm and mitochondria under both normal and oxidative stress conditions with fluorescent probes. They found that dapagliflozin significantly mitigated the increase in ROS within the cytoplasm and mitochondria of proximal TECs during oxidative stress conditions and altered Ca^2+^ dynamics (136). Furthermore, dapagliflozin reduced macrophage infiltration in the kidneys of db/db mice, resulting in reduced expression of inflammatory cytokines and genes associated with oxidative stress, including monocyte chemoattractant protein-1 (MCP-1) and osteopontin (137).

In the clinical setting, initially introduced for the therapy of T2DM, SGLT2 inhibitorshave subsequently exhibited positive therapeutic effects in numerous trials targeting liver and kidney outcomes. Research have show that SGLT2 inhibitors can markedly decrease proteinuria, slow the progression of renal function decline, and decrease liver fat content; they also improve serum transaminase levels (138–140). A meta-analysis evaluated liver function and structure in patients with type 2 diabetes, comparing the effects of SGLT2 inhibitors with those of placebo or other oral hypoglycemic agents. The findings revealed that SGLT2 inhibitors effectively reduced serum levels of alanine transaminase (ALT), aspartate transaminase (AST), and γ-glutamyl transpeptidase (141). Furthermore, several large-scale cardio-renal outcome studies have demonstrated the renal advantages of SGLT2 inhibitors in patients with T2DM (142–146).

### GLP-1 receptor agonists

GLP-1 receptor agonists (GLP-1RAs) are increasingly recognized as novel therapeutic agents for the treatment of type 2 diabetes. Their significant effects on metabolic regulation, weight management, and cardiovascular and renal protection have led to a growing focus on GLP-1RAs in the context of MLKD. Investigations have revealed that liraglutide reduces oxidative stress by increasing SOD levels. This includes decreasing serum malondialdehyde levels, MCP-1 expression, and NF-kB levels, while also inhibiting endogenous inflammatory responses (147, 148). Additionally, liraglutide enhances heme oxygenase-1 concentration in human serum, indicating a possible improvement in antioxidant capacity (149). Furthermore, semaglutide elevates serum and hepatic SOD levels in HFD-induced MASH mice, thus preventing hepatic lipid accumulation, exhibiting anti-inflammatory effects, and improving mitochondrial architecture by reducing mitochondrial swelling and promoting more ordered cristae (150). In the kidneys, exenatide reverses the downregulation of Sirt1 in FFA-induced TECs and prevents the increase in ROS. This intervention prevents declines in MMP and attenuates mitochondrial apoptosis (151).

In the early stages of MASLD treatment, GLP-1RAs as well as glucose-dependent insulinotropic polypeptide (GIP) and GLP-1 dual receptor agonists are commonly utilized (152). Although the specific benefits of these agents for liver fibrosis remain unclear, they indirectly enhance liver health by promoting weight loss, thus reducing hepatic fat and inflammation (153). The CGH-LiNASH study confirmed that liraglutide effectively reduces the weight of obese adult patients with MASLD and improves liver steatosis and hepatocyte apoptosis (154). In relation to kidney health, GLP-1RAs have been demonstrated to slow the progression of diabetic kidney disease, as evidenced by the LEADER study (155). The research investigated the impact of liraglutide on 23% of CKD patients, revealing a reduction in the risk of renal failure by approximately 25%, alongside decreased serum creatinine levels, reduced mortality risk due to kidney disease, and lower incidence of macroalbuminuria (155). Other GLP-1RAs, such as semaglutide, dulaglutide, efpeglenatide, lixisenatide, and the dual receptor agonist tirzepatide, have also demonstrated similar effects on macroalbuminuria (156).

### PPAR-γ agonists

PPAR-γ is a nuclear receptor that regulates lipid metabolism and glucose homeostasis. Thiazolidinediones (TZDs), which are categorized as PPAR-γ agonists, have been explored for their potential use in managing MLKD. As discussed earlier, TZDs, including pioglitazone, have the capacity to stimulate mitochondrial biogenesis and improve mitochondrial function (157). Their mechanism of action involves the induction of PGC-1α, a key transcriptional coactivator that regulates mitochondrial biogenesis, oxidative phosphorylation, and fatty acid oxidation (158). They have demonstrated significant therapeutic potential in MLKD, and future research will further explore their clinical applicability in different disease states.

A systematic review indicated that pioglitazone treatment improved individual histological scores for MASH compared to placebo and increased the remission rate for MASH (159). Pioglitazone (45 mg/day for 72 weeks) was superior to placebo in improving fibrosis scores in MASH patients, particularly those with T2DM. In the context of DKD, a retrospective cohort study involving 742 patients revealed that pioglitazone did not significantly reduce the risk of composite renal endpoint events, although a non-significant reduction in proteinuria was noted (160). Further investigation is essential to thoroughly elucidate the therapeutic potential and clinical significance of pioglitazone in individuals with CKD.

### Mesenchymal stem cells

MSCs are multipotent progenitor cells capable of self-renewal and differentiation. The versatility in sourcing of MSCs provides a strong basis for applications in cell-based therapy and regenerative medicine (161). Owing to their self-renewal, differentiation potential, regenerative, and immunomodulatory properties, MSCs have garnered attention as a promising therapeutic approach for the management of hepatic and renal disorders.

In recent years, the application of bone marrow-derived mesenchymal stem cells (BM-MSCs) through transplantation has been utilized in various mouse models of MASLD. Studies have shown that BM-MSCs can effectively mitigate hepatic steatosis, inflammation, and fibrosis. They also enhance hepatic glucose and lipid metabolism, boost mitochondrial function, and decrease liver injury and apoptosis (162). BM-MSCs have the ability to transfer functional mitochondria to injured tissues via mechanisms like tunneling nanotubes, extracellular vesicles, and cell fusion, which subsequently enhances tissue repair (163). In MASLD mouse models, mesenchymal therapy has been shown to improve mitochondrial dysfunction through mitochondrial transfer, stimulate mitochondrial function and diminish calcium accumulation in steatotic hepatocytes, thereby alleviating hepatic steatosis (164). In the renal context, BM-MSCs promote the activation of endothelial nitric oxide synthase (eNOS) through phosphorylation (165), enhance mitochondrial function (166), and decrease the production of ROS, markedly suppressing oxidative stress and enhancing renal function (167). In DKD mouse models, BM-MSCs induce an anti-inflammatory phenotype in renal macrophages through mitochondrial transfer, which ameliorates kidney damage. This effect depends on PGC-1α-mediated mitochondrial biogenesis and PGC-1α/TFEB-mediated lysosomal autophagy (168).

In clinical setting, a post-hoc analysis of a prospective clinical trial (NCT02302599) demonstrated that umbilical cord-derived MSCs (UC-MSCs) achieved a liver fat reversal rate of up to 45.45% in patients with T2DM complicated by MASLD after 20 weeks of treatment, significantly reducing body mass index (BMI), fasting blood glucose levels, triglycerides, and ALT levels (169). Additionally, an 18-month single-arm safety follow-up study (NCT02195323) indicated that the injection of a single dose of autologous MSCs in patients with CKD was safe and well-tolerated, although no statistically significant changes in renal function were observed (170). A randomized clinical trial by Perico et al. (NCT02585622) demonstrated that, compared to placebo, cell therapy significantly slowed the decline of eGFR over 18 months (171). Future studies should further investigate the mechanisms of MSCs, optimize treatment protocols, and address potential challenges in clinical applications to fully harness their therapeutic potential in the treatment of MLKD.

While BM-MSCs offer promising therapeutic potential, several challenges remain. These include issues related to graft rejection, limited cell engraftment, and difficulties in scaling up for clinical use. For instance, despite the immunomodulatory properties of MSCs, the risk of graft rejection cannot be entirely ruled out, especially in allogeneic settings (172). Additionally, achieving sufficient cell engraftment and long-term survival of transplanted cells remains a significant challenge, which can limit the therapeutic efficacy of MSCs (173). These challenges highlight the need for continued research and development to optimize MSC-based therapies.

## Conclusion and future perspectives

An increasing amount of both experimental and clinical evidence indicates that the shared pathogenic pathways of mitochondrial dysfunction in MLKD have been extensively established. The intricate interplay among oxidative stress, mitochondrial biogenesis, dynamics, and mitophagy in both the liver and kidney is crucial for the progression of MLKD, and these processes are tightly interconnected. Several therapeutic agents, such as antioxidants, SGLT-2 inhibitors, GLP-1RAs, PPAR-γ agonists, and MSCs, have shown promise in treating MLKD by modulating mitochondrial function. It is mentioned that the therapeutic benefits of these agents are not solely attributable to these mechanisms. Antioxidants and SGLT-2 inhibitors have been shown to reduce oxidative stress and inflammation (174), while GLP-1RAs and PPAR-γ agonists have demonstrated immune-modulatory properties (175). MSCs also exhibit anti-inflammatory effects through cytokine modulation and immune cell regulation (176). These additional mechanisms underscore the multifaceted nature of these agents’ therapeutic benefits and highlight their potential for addressing complex pathophysiological processes in MLKD.

Additionally, the mechanisms underlying MASLD-induced kidney injury and the liver-kidney crosstalk remain underexplored. Elucidating the molecular signaling pathways, cellular interactions, and shared inflammatory mechanisms between the liver and kidneys will be crucial for advancing our understanding of the association between the liver and kidneys. Further research is needed to elucidate these mechanisms and develop potential new therapies for MASLD-associated CKD.
